# Growing intergenerational resilience for Indigenous food sovereignty through home gardening

**DOI:** 10.5304/jafscd.2019.09b.018

**Published:** 2019-12-21

**Authors:** Rachael Budowle, Melvin L. Arthur, Christine M. Porter

**Affiliations:** Haub School of Environment and Natural Resources; University of Wyoming; 1000 E. University Avenue; Laramie, WY 82071 USA; Department of Kinesiology & Health, College of Health Sciences; University of Wyoming; 1000 E. University Avenue. Dept 3196; Laramie, WY 82071 USA; Growing Resilience Principal Investigator; Division of Kinesiology & Health, College of Health Sciences; University of Wyoming; 1000 E. University Avenue, Dept 3196, Laramie, WY, USA

**Keywords:** Intergenerational Resilience, Food Sovereignty, Community Resilience, Social-Ecological Systems, Sovereign Storytelling, Growing Resilience, Indigenous, Historical Trauma

## Abstract

As a community-based participatory research project designed to promote health and wellbeing, Growing Resilience supports home gardens for 96 primarily Eastern Shoshone and Northern Arapaho families in the Wind River Reservation, located in Wyoming. Through analysis of data from two years of qualitative fieldwork, including stories told by 53 gardeners and members of the project’s community advisory board in talking circles and through our novel *sovereign storytelling* method, we investigated if and how these participants employ relationships, knowledge, and practices across generations through home gardening. We find that participants describe home gardening within present, past, future, and cross-generational frames, rooted in family relationships and knowledge shared across generations. Our analysis of these themes suggests that gardening provides families a means to transmit resilience across generations or, as we call it here, *intergenerational resilience*. We conclude by discussing intergenerational resilience as a culturally specific mechanism of social-ecological community resilience that may be particularly relevant in Indigenous movements for food sovereignty.

## Introduction

The international peasant movement Via Campesina defines food sovereignty as “the right of peoples to healthy and culturally appropriate food produced through ecologically sound and sustainable methods, and their right to define their own food and agriculture systems” (Via Campesina, 2007, para. 3). This vision of food sovereignty also includes gender, labor, and Indigenous rights (McMichael & Porter, 2018). Though such a universal rights–based perspective does not draw on Indigenous worldviews of collective wellbeing and connections to the environment, liberatory power transfers in food systems are essential to food sovereignty, including in Indigenous contexts (Carney, 2012; Coté, 2016; Kamal, Linklater, Thompson, Dipple, & Ithinito Mechisowin Committee, 2015; Patel, 2009). More specifically, Indigenous food sovereignty requires moving beyond rights to focus on the “cultural responsibilities and relationships that Indigenous peoples have with their environment. It also requires examining the efforts being made by Indigenous communities to restore these relationships through the revitalization of their Indigenous foods and ecological knowledge systems as they assert control over their own wellbeing” (Coté, 2016, p. 2).

Growing Resilience, a community-based participatory research (CBPR) project, is part of one such effort toward Indigenous food sovereignty. The project provides installation and maintenance support for home food gardens to 96 families living in the Wind River Reservation (WRR) in Wyoming. Eastern Shoshone and Northern Arapaho people in the WRR experience enormous health disparities, including obesity, diabetes, and dying up to 30 years earlier on average than White people in Wyoming (Porter, Wechsler, Hime, &, Naschold, 2019; Williams, 2012). Growing Resilience aims to reduce those disparities, support local food sovereignty leadership, and evaluate health impacts of home gardens using a randomized controlled trial design (Growing Resilience, n.d.; Porter, Wechsler, Naschold, Hime, & Fox, 2019).

Growing Resilience originated conceptually in 2011 when the Indigenous-led, WRR community organization Blue Mountain Associates participated as one of five community-based organizations in the Food Dignity project. Food Dignity was a CBPR project that investigated and supported how community-based organizations across the country work toward food justice (Porter, Woodsum, & Hargraves, 2018; Sutter, 2018) and its “close cousin,” food sovereignty (McMichael & Porter, 2018). Blue Mountain Associates found that community interest in food gardening exceeded their resources to support new gardens.^1^ Thus, following a pilot phase, Growing Resilience partners secured funding from the National Institutes of Health (NIH) to provide and evaluate health impacts of home gardens, with Blue Mountain Associates providing the garden support. Additionally, the community advisory board that guides the project, along with other project partners, desired to document much more than quantifiable, individual health outcomes from the collaboration.

Accordingly, we pursued qualitative inquiries into processes and outcomes of wellbeing and resilience through home gardening experiences. In this paper, we investigated if and how participants in Growing Resilience build relationships, knowledge, and practices across generations through gardening. This generational focus first emerged during our field experience with Blue Mountain Associates and the community advisory board, with their recollections of past home gardening in WRR and in their own family histories. We then explored what we call *intergenerational resilience* embedded in participants’ stories, based in frameworks of Indigenous food sovereignty and community resilience.

## Literature Review

We grounded our investigation of resilience across generations through home gardening in a review of approaches from Indigenous food sovereignty, socio-ecological community resilience, and Indigenous resilience.

### Indigenous Food Sovereignty

Foundational food sovereignty perspectives are based on rights and require cultural diversity and appropriateness (Via Campesina, 2007). Indigenous perspectives on food sovereignty also focus specifically on relationships, including with other people, ancestors, living things, and the land, centralizing individual and community wellbeing (Coté, 2016). The processes of decolonization and self-determination, based not necessarily in the state but in terms of struggles for collective autonomy, are integral to Indigenous food sovereignty (Grey & Patel 2015).

An Indigenous kincentric ecology, one that interactively connects people and other ecosystem elements in common ancestry or kinship, further explains the relational basis of many Indigenous food sovereignty efforts (Salmón, 2000). For Nuucha-nulth people in British Columbia, for example, food sovereignty emerges from decolonization and reclamation of traditional lands and control of fisheries. Coté (2016) describes how those efforts are based in iisak,^2^ or respect, and ancestral knowledge that guide an interconnected web of relationships with other people and all living things. For the O-Pipon-Na-Piwin Cree people in Manitoba, food sovereignty—including hunting, wild food gathering, and gardening—finds a basis in wechihituwin, “any means of livelihood that is shared and used to help another person, family, or the community” (Kamal et al., 2015, pp. 565–566). Their Food from the Land program supported harvesting wild foods, sharing gathered food in the community, and elder-facilitated gathering classes for youth. Through storytelling, elders explained how sharing and youth training based in wechihituwin informed their distinctly relational approach to decolonization (Kamal et al., 2015).

Grey and Patel (2015) draw on Adelson’s (2000) work with Cree people highlighting their concept of miyupimaatisiium or “being alive well.” Based in connections to the land and access to traditional food, they apply the concept to Indigenous food sovereignty beyond the Cree culture. They further suggest that food provides a particularly resonant way to understand wellbeing and shared relationships between Indigenous people and land.

### Social-Ecological and Community Resilience Frameworks

The centrality of relationships and interconnections between people and ecosystems in Indigenous food sovereignty overlaps with social-ecological systems and community resilience perspectives. Emerging from ecology, these perspectives attempt to blend coupled human and natural systems into one framework (Berkes & Folke, 1998; Westley, Carpenter, Brock, Holling & Gunderson, 2002). Striving for resilience—the ability of social and ecological systems to adapt to change and disruption—is a hallmark of these approaches (Folke, 2006; Walker, Gunderson, Kinzig, Folke, Carpenter, & Schultz, 2006).

In these frameworks, food systems resilience is “capacity over time of a food system and its units at multiple levels to provide sufficient, appropriate and accessible food to all, in the face of various and even unforeseen disturbances” (Tendall et al., 2015, p. 19). Through case studies of food systems in southern states, Worstell & Green (2017) developed an index based on eight qualities of resilient food systems, including local self-organization. King (2008) suggests that alternative agricultural practices, including community gardens, can benefit from social-ecological systems approaches to build resilient communities and ecosystems. In the Czech Republic, survey data indicate that self-provisioning of food through home gardens contributes to resilience beyond immediate economic benefits to strengthen social ties through food sharing practices (Jehlička, Daněk, & Vávra, 2018).

Social-ecological systems resilience and food sovereignty share similarities, including a shift in attention to local knowledge and governance, a focus on both human and natural elements, and a process orientation (Walsh-Dilley, Wolford, & McCarthy, 2016). Social-ecological systems resilience, however, receives criticism for an overemphasis on formal institutions as opposed to human activity and agency, approaches that are overly functionalist, inattention to specific cultural and historical contexts, universal frameworks that do not allow for consideration of social diversity within and between communities, and notably, inattention to power (Brown & Westaway, 2011; Cote & Nightingale, 2012; Fabinyi, Evans, & Foale, 2014, 2014; Olsson, Jerneck, Thoren, Persson, & O’Byrne, 2015). Lessons from food sovereignty add a sharper focus on power and social justice to these resilience frameworks (Walsh-Dilley et al., 2016).

Integrated community resilience, emerging from social-ecological systems, psychology, and community development perspectives, provides a more fitting framework for Indigenous food sovereignty than broader social-ecological approaches (Berkes & Ross, 2013). As “the existence, development, engagement of community resources by community members to thrive in an environment characterized by change, uncertainty, unpredictability, and surprise” (Magis, 2010, p. 402), community resilience includes characteristics of people-place relationships, social networks, knowledge and skills, and values and beliefs (Berkes & Ross, 2013). Rather than a focus on systems, community resilience focuses on community in place—real people engaged in physical locations and relationships (Amit & Rapport, 2002; Berkes & Ross, 2013; Cohen, 1985). Following the ample literature on the problematic notion of a monolithic ‘community’ (see, e.g., Agrawal & Gibson, 1999; Dove, 2006; Fabinyi et al., 2014; Titz, Cannon, & Krüger, 2018), community resilience and critiques of broader social-ecological systems perspectives recognize that resilience responses vary across cultures and contexts (Berkes & Ross, 2013; Cote & Nightingale, 2012; Leslie & McCabe, 2013).

While more fitting for people’s connections to land and place in Indigenous food sovereignty than broader social-ecological systems perspectives, community resilience requires further empirical research, including specific research into how communities respond to change and draw on social memory from the past to inform responses in the present and into the future (Vaneeckhaute, Vanwing, Jacquet, Abelshausen, & Meurs, 2017). Therefore, we finally turn to Indigenous and other perspectives on resilience that highlight relational and multigenerational responses to drastic changes and trauma.

### Indigenous Resilience

Indigenous resilience highlights individual, family, and community-level perseverance and thriving despite historical trauma and ongoing structural violence (Kirmayer, Dandeneau, Marshall, Phillips, & Williamson, 2012; Kirmayer, Gone, & Moses, 2014). Historical trauma “is the legacy of numerous traumatic events a community experiences over generations and encompasses the psychological and social responses to such events” (Evans-Campbell, 2008, p. 320). A deep literature explains how trauma is physically, mentally, and spiritually transmitted across generations as a result of colonization (see, e.g., Brave Heart & DeBruyn, 1998, Evans-Campbell, 2008; Fast & Collin-Vézina, 2010). That trauma both challenges and requires resilience of Indigenous people (Fleming & Ledogar, 2008), for example, as indicated by varied responses to the trauma inflicted by boarding schools (Colmant, Schultz, Robbins, Ciali, Dorton, & Rivera-Colmant, 2004; Wexler, 2006).

Ethnographic research into Maidu people’s efforts for Indigenous representation on a stewardship council in California suggests that recognition of historical trauma is a prerequisite for healing and action (Middleton, 2010). Anthropological perspectives explain trauma responses as relational, with healing occurring through connection with others, including in ways that often have little to do with the traumatic events themselves (Lester, 2013). Previous research examining the historical trauma response of Palestinian refugees employs the phrase “intergenerational resilience” in ways akin to our approach here, including elders sharing cultural stories with children and learning for everyday resistance (Atallah, 2017). Denham’s (2008) ethnographic research with Nez Perce families demonstrates that they transmit resilient strategies through strength-based family narratives about historical trauma. Similarly, the Roots of Resilience mental health project suggests that—much like community resilience and Indigenous food sovereignty—Indigenous perspectives extend resilience to the community through stories that provide connection between individuals, families, the environment, ancestors, and other living things from the past to the present (Kirmayer et al., 2012).

Perspectives from Indigenous food sovereignty, social-ecological community resilience, and Indigenous and generational resilience are relational, including relationships between people and their environment over time. Together, the bodies of literature above provide a foundation for our investigation of the relationships, knowledge, and practices that home gardeners employ across generations in Growing Resilience. Through this review, we also uncover opportunities to extend concepts of the generational transmission of resilience and provide further empirical investigation of community resilience and Indigenous food sovereignty.

## Methods

The entire Growing Resilience project, including this qualitative inquiry, adopts a community-based participatory research approach (CBPR). CBPR is a variant of action research, which engages community and academic co-researchers in research design, implementation, and dissemination with an explicit, ideal goal to enhance equity and promote social transformation through the research process (Greenwood & Levin, 2007; Israel, Eng, Shulz, & Parker, 2013). Additionally, participatory action research methods may not only assess but actively build community resilience through the research process itself (Ross & Berkes, 2014; Vaneeckhaute et al., 2017). These ideals, of course, are not always actualized, including within food justice-focused research (see e.g., Bradley & Herrera, 2015; Porter & Wechsler, 2018; Woodsum, 2018). Building from this CBPR approach, we pursued this inquiry with a broad ethnographic methodological orientation through which we conducted two years of fieldwork from 2016 to 2018. With a commitment to ethnographic interpretation of meaning and “thickness” of description and analysis to honor the lived experiences of the research participants, we wove together multiple interrelated methods, including participant observation and other qualitative methods (Geertz, 1973; Ortner, 2006), which we describe in detail below. In total, our analysis draws on stories from 53 people who are directly involved with Growing Resilience.

We obtained Institutional Review Board approval for Growing Resilience and all qualitative research included therein prior to beginning this inquiry. Additionally, we sought and obtained approval for Growing Resilience research from both Eastern Shoshone and Northern Arapaho Tribal Business Councils. The Growing Resilience Community Advisory Board, consisting of Eastern Shoshone, Northern Arapaho, and other Sovereign Nation members, has provided guidance and approval for all aspects of this research from research question conceptualization to data collection to dissemination in this present form. We obtained written informed consent for qualitative research participation from participants during their initial quantitative health data collection, and we reviewed consent with them again prior to participation in qualitative research.

We, the co-authors, consist of a White woman, who was an anthropology doctoral candidate at the time of data collection, Budowle; a Northern Arapaho man and research scientist, Arthur; and another White woman, who is the principal investigator of Growing Resilience, Porter. Budowle and Arthur led the development and implementation of this research with support and input from Porter. We have relied on our respective outsider and insider statuses in the WRR community, and we collaborated on gathering data, developing research questions, and analyzing and interpreting data. Our approach also reflects Arthur’s commitment to research that honors and transmits ancestral and present-day Indigenous stories in WRR communities (Arthur & Porter, 2019; Bradley, Gregory, Armstrong, Arthur, & Porter, 2018). Accordingly, stories—which can promote both individual and community resilience in Indigenous contexts—provide our primary data source for the present research (Kirmayer et al., 2012).

### Data Collection

While fieldnotes from participant observation inform our analysis in a general way (DeWalt & DeWalt, 2011; Sanjek, 1990), we rely primarily on two main sources of stories for more detailed analysis and coding: talking circles and a novel methodological approach we developed during the research process, which we call *sovereign storytelling*. Participation in talking circles and sovereign storytelling was optional and additional to participation in the overall Growing Resilience project and associated quantitative health data collection. Our purposive sample included any adult participant who was randomly assigned to the treatment, or gardening, condition (i.e., not a participant randomly assigned to the control condition who had not yet participated in gardening) and who wished to participate (Guest, 2015). We invited participants to join talking circles and share stories at health data collections, during gardening workshops, and through direct contact by phone. Only adult participants were eligible to formally participate in qualitative research; however, children frequently joined in and around sovereign storytelling informally, particularly during onsite garden visits, through photo stories, and by creating artwork-based stories with adult family members.

As suggested by the community advisory board, talking circles took the form of culturally responsive focus groups (Rodriguez, Schwartz, Lahman, & Geist, 2011). Accordingly, we observed locally appropriate customs for group discussion through talking circles, in which participants pass a talking stick and allow each person to talk uninterrupted while they hold the stick and until passing it to the next person who wishes to speak. A community elder made the talking sticks specifically for use in Growing Resilience qualitative research, and another elder blessed the talking sticks prior to use in our talking circles. We conducted two talking circles (*N*=14; *N*=11) totaling 25 participants. We also facilitated a talking circle with community advisory board members (*N* =6) who asked for an opportunity to share their stories about the project and gardening (Bowers, Harris, Harris, Lone Fight, & Weed, 2019). We prompted participants with a digital storytelling video focused on gardening in the WRR produced by a co-investigator at Blue Mountain Associates during the Food Dignity project (Potter, 2015).

After completing two participant talking circles, we implemented sovereign storytelling as a way to infuse participant choice and voice into our qualitative methodology, given that the colonizing process of research, including food justice research and CBPR, fails to provide the “means for research participants to shape or respond to how they are represented” (Bradley & Herrera, 2015, p. 104). Sovereign storytelling seeks to purposefully allow participants a say in that representation and to highlight Indigenous stories as an active way to contribute to the decolonization of research (Smith, 2012). We asked participants as individuals or families if and how they would like to tell their stories to us as researchers and if and how they would like to share those stories with the greater community in their own voices. Participants could opt to tell their story through single or multiple methods. All participants consented to sharing their stories for research, including as presented here. Some also opted to share their stories directly, including with the WRR community.

We provided a brief menu of potential storytelling methods to participants (although we invited participants to engage in storytelling methods other than those suggested in the menu):

- Participating in an interview, as an individual or as a family;
- Participating in a group talking circle;
- Talking informally about their garden during a home garden visit;
- Taking pictures of their garden and writing a bit about the photos in captions;
- Keeping a garden journal and sharing some or all entries;
- Making art about their garden (e.g., poem, story, sculpture, drawing, beading); and
- Making a short film about their garden.

In total, 22 participants engaged in storytelling, resulting in 15 unique stories, as several people opted to tell their stories as couples or families. Participants selected a variety of storytelling methods, including interviews, home garden visits, photos with captions, videos, and artwork.

Using a person-centered approach that allows for illumination from individual experience to the broader community and sociocultural context (Levy & Hollan, 2015), we asked participants two key questions for both talking circles and sovereign storytelling:

1. What does the gardening experience provide/mean for you and your family?
2. What does the gardening experience provide/mean for your community?

We generated verbatim transcripts of participants’ stories from talking circles, interviews, garden visit conversations, videos, and photo captions. While not included in our coding scheme, which we detail below, holistic understandings of stories, artwork, photos, and fieldnotes from health data collections, garden visits, garden workshops, Growing Resilience open houses and celebrations, community advisory board meetings, and various planning meetings with Blue Mountain Associates and our academic research team more generally inform our analysis.

### Data Analysis

After correcting transcripts, we used Dedoose software to aggregate and code our data (Dedoose, n.d.). We generated initial coding themes deductively, shaped by Growing Resilience research questions about mechanisms of health and wellbeing related to gardening (Bernard, 2006). Simultaneously, we used a grounded theory approach, allowing themes to emerge from the data (Glaser & Strauss, 1967; Strauss & Corbin, 1990). This combined deductive and inductive approach provided direction for our analysis while also allowing other important themes to emerge (Miles & Huberman, 1994). Along with our time in the field, this analysis prompted us to investigate generational and familial relationships. We each independently developed initial codes from our first talking circle transcript. Then we collaborated to refine our coding scheme as an academic research team before finally checking it with the community advisory board. The inclusive code-generation process provided validity and reliability according to standards for ethnographic research in our analysis (LeCompte & Schensul, 2012; Trotter, Schensul, & Kostick, 2015).

While Budowle and Arthur independently coded all data, Budowle served as the primary coder for this analysis. In this research, we employed a joint coding approach less as a means to quantitatively calculate interrater reliability, but more to use Arthur’s codes and coding as a general check against Budowle’s, given his deep familiarity with the research context. This approach is in keeping with the team-based methodology that we previously described and allowed us to focus on deep qualitative insights and the extension of community resilience and food sovereignty frameworks relevant to our grounded approach (Bernard, 2006; LeCompte & Schensul, 2012; Yin, 2009).

Our entire Growing Resilience qualitative inquiry examined broad mechanisms of resilience, health, and wellbeing associated with home gardening. After deductively coding for these mechanisms, we identified several codes potentially relevant to themes of family and generations. Passages identified with these codes represented 44% of our overall dataset. After removing passages coded as ‘gardening practices,’ which emerged as the most frequent code in our overall dataset, wherein participants discussed actual or planned gardening in a highly technical or practical way, excerpts related to family and generations made up 66% of our coded passages. The prevalence of these codes in our overall dataset suggests that while participants discussed gardening practices, food, and health, they readily contextualized those discussions in terms of family and generations.

Accordingly, we specifically narrowed our scope in this research to family relationships, which we define as those relationships involving children, grandchildren, parents, grandparents, and other broad familial and generational relationships across time (as opposed to other social, nonfamilial relationships). This yielded a dataset of over 200 unique excerpts. After further analysis of these excerpts, the following intergenerational themes emerged, around which we organize the presentation of findings below: *family; togetherness; teaching and learning; parents, grandparents, and past generations; knowledge and traditions; historical trauma; perseverance and expansion; children and grandchildren; visons and hope;* and *shared knowledge and memory-making*. Finally, we checked the validity of our themes with other members of the Growing Resilience team (Glaser & Strauss, 1967).

## Results

We use an analytical framework of *present*, *past*, *future,* and *cross-generational* to organize the above themes. These frames serve as linguistic representations of time that allow us to locate social meaning within their bounds (Goffman, 1974), even though, as we discuss below, a cyclical representation of these frames may be more fitting for an Indigenous concept of time.

### Present

Participants most frequently described their garden experiences through present familial relationships and practices, including themes of *family; togetherness; and teaching and learning.*

### Family

Gardeners often described their present experience with family in general ways. Within Growing Resilience, households frequently consisting of multiple generations participated in the garden and health data collection together, and the family experience was a focus for the study and for participants. Many discussed their gardening experience in relation to not only the family members participating in the project, but also to those not participating and to extended family members. One participant described her eagerness to support her father with gardening as a key reason for participating in the project: “Because my dad always talked about ‘oh we need to get a garden,’ so I was like, okay, this is our chance. I’m going to get him involved.”

While participants generally talked about their entire families, including parents and siblings, they heavily focused on their children and grandchildren in relation to their gardens. Many described how children helped with various stages of gardening from planting to harvest and took ownership over specific tasks within the garden. Participants additionally made connections between children and the growing process, for example:

They’re interested in something that you grew, and you’re trying to tell them that you’re growing it for them. That’s what you want: to try to just grow stuff for them, try to get their own little garden growing for them.

Growing it, and if you got kids, it’s the same way—you’re growing them up too.

Notably, the design of the pilot version of Growing Resilience included only adults in the health data gathering portion of the project. However, the participating families and advisory group at the time said it was imperative to include children not only in the gardening, but also the health data gathering in the full-scale project. Similarly, children were a focus for community advisory board members, many of whom garden and some of whom participated in pilot iterations of the study. They described healthy families as a primary motivation for serving on the board and noted family benefits as a gardening outcome:

And I see their light. Their whole families light up. I mean their kids, you wouldn’t think young kids would get into it, but they do get into it.

The kids see the sprouts coming up, and it’s so exciting to them to know that these are growing. Then when they can pick it, you tell them “go get me two squash out of the garden,” and they’ll run out and bring them in, “we found them, we found them!” And they watch to see—it’s something, it’s life. I think that the families that are involved will continue.

### Togetherness

Many participants talked about how gardening brought their families together in the present, involving their children in something positive and often resulting in a sense of accomplishment and pride. Participants noted strengthened relationships, including spousal, parent-child, and across the family, for example:

I have eight children and my husband here, and we really love the garden. It helped us as a family, to come together. … I think it really helped me with the bonding with the children and with my husband. And, it meant a lot to us as a family.

My kids didn’t know before this year of us having our own garden, so I was really proud that they were right there with me, hands on doing it, getting dirty, and not even complaining. Usually they notoriously complain, but this time they actually really looked forward to it. And, it made me really proud that they wanted to know how to grow their own food.

It makes us all really excited together, to see that we’ve done this together. Especially when we get something like the zucchini, and it’s like, “look we did it; we’ve scored!” It’s something we all did together, and it’s for us.

One participant noted the potential for gardens to strengthen family relationships for other people:

If more people got a garden, it’d be better for their families, because they’re all involved in it. My kids really enjoy it. My little guy, he finally got to where he could start getting involved this summer…. I think if people get their children involved, then it will stop on some of the violence later on, because they’re more involved with what’s going on at home than what’s going on out here.

Bringing families together through gardening similarly emerged for the community advisory board:

It’s really been a pleasure to see how much it’s helped our community and the people who are actually growing it, because it brings your family together. That’s your livelihood—a long time ago if you didn’t have a garden, you didn’t eat. But, they’re not seeing it that like that; it’s more bringing their families together, everybody working together.

A co-investigator with Blue Mountain Associates reported that a participant thanked her for supporting her garden and bringing the family together. The participant shared that instead of sitting inside all doing different things, her family now sits outside by the garden, watching the sun set while talking and answering the children’s questions.

### Teaching and learning

Many participants described the garden as a mechanism for them to teach their families and for children to learn, for example:

It’s awesome, because now I’ve learned so much—he’s learned a ton—and now we can teach our kids. I taught my daughter-in-law. She had a little garden at their place this year…. It’s kind of a together thing. It brings everybody together, because I can bring someone out and “look what I learned today!” and then I teach them to do it, and now everybody knows. And then everybody’s excited when stuff comes from the garden, and we get to eat it.

One married couple explicitly connected their children’s time in the garden to learning healthy eating and cooking skills. They described how harvesting from the garden reminded them that food does not have to come from a store, which “opened our kids’ eyes too.” They attributed their children’s recently diminished food aversions to the garden, explaining that “getting them involved and actually feeding them the food that we’re getting kind of opened, broadened their horizons of food.” With their son learning to cook food from the garden, despite having never demonstrated interest in cooking previously, his mother hoped cooking “will take him somewhere, eventually.”

Beyond gardening and food knowledge, participants described how the garden facilitated learning around responsibility, self-reliance, and empowerment for themselves and their families:

It may be a chore, but one day they’ll realize that this thing needs its nourishment too, and they got to give their time to this garden, so it’ll grow. And, it takes patience and time; it’s not just something you could hope it’ll live on its own, or, “it’ll be okay, it was really hot today. I’ll just worry about it tomorrow,” and then say that the next day and the next day. It takes your time and attention every day. You have to put effort into it to get it going. And, they’re learning that.

It’s instilling in our kids, showing them that we’re able to do this ourselves instead of relying on the stores for their produce and waiting. And teaching them, empowering them that really, they’re able to grow their own food.

I think it’s been a really good experience, because not a lot of people know how to grow stuff. It’s easier just going to the grocery store. One of the essential things about life is growing stuff. If you can grow a garden, you can do almost anything.

### Past

Participants connected their current experience to gardening in the past. *Parents, grandparents, and past generations; knowledge and traditions;* and *historical trauma* themes are organized within the *past* frame.

### Parents, grandparents, and past generations

Many participants recalled that their families—particularly parents and grandparents—maintained gardens in the past, often out of necessity. One participant noted that his mother’s large garden took time to establish, but she was able to improve it over time to grow large quantities of food, including corn. He explained, “she grew up really poor right over here just on the river, and they grew their own stuff.” Others described parents and grandparents who canned and preserved food from large gardens to last throughout the year. Some even recalled gardening or eating from the garden as children:

When I was growing up my folks had a big old huge garden, and we never went to town, bought candy or anything. When we got hungry, we’d just run out to the garden and get us a turnip or carrots. Then we’d take off again. We’d go cruising wherever we were going, go back to the river to swim or horseback. We always had something to do. But our garden—we just raided in our garden all the time; it was good. We had lots of corn and these types of food at the table. And my grandmother had a huge old garden. My aunt and I used to have to always be hoeing it and watering it.

Some participants and members of the community advisory board recalled a more comprehensive use of gardens in the WRR and generations past. One remembered that the entire community kept large gardens and used cellars to store food over winters. A participant noted that the act of gardening today is explicitly connected with practices in the past:

Basically, planting them, and they do the same thing they did a long time ago. They still put the seed in the ground, took care of it, nourished it, and give it the love it does, and it’ll come up the way it needs to. And it connects by just that feeling of taking care of it, and when you do get all the vegetables and stuff from it and you can benefit from it.

### Knowledge and traditions

Beyond specifically recalling family members and past generations who gardened, participants described past knowledge and traditions involved with gardening and food. Participants frequently described gaps in past knowledge and skills as an obstacle to their current gardening. A couple explained how, despite their lingering interest, their family had stopped gardening as a result of their grandmother’s death and that most family gardening knowledge and commitment largely died along with her. Another participant described knowledge as “dormant” and noted that their family relied on living grandparents to access gardening knowledge from the past. One participant explained how challenging gardening was due in part to gaps in generational knowledge: “It was really hard for us to know how to do a garden. We’ve never done that before in our lives. We have moms that know how to do them very well, but we didn’t know how.”

While participants noted the challenges with knowledge gaps, many expressed a desire to reclaim lost skills and traditions relevant to gardening and food preservation from earlier in life and previous generations for themselves and the broader community today:

I think it’s a good experience. People are getting back to ground roots, stuff we grew up on when we were young. I learned how to plant and maintain a garden and take the veggies out and use them.

I never knew how to go to the grocery store growing up. We ate everything canned. And now, I’m trying to learn how to do all that stuff after all these years. It is a lot healthier. People were healthier back then. My grandmother lived to be 95, and I think puttering in the dirt was probably the best thing. And I found out that when I have a lot of stress, I go putter in the dirt, and that actually, puttering today might be a good idea.

I’ve seen some people that have maybe had a garden before, but it just kind of went away, by the wayside. Why can’t you build it back up?

### Historical trauma

Some participants and community advisory board members explained the loss of gardening from previous generations in terms of long generational processes of historical trauma leading to present outcomes. A participant equated pervasive drug and alcohol use in the WRR with gardening fading over time. For that reason, he wished he had an opportunity to garden earlier in life: “I wish that was started a long time ago, when I was a little guy. I would’ve been already doing this.”

Others made explicit connections between boarding schools and the loss of gardening—and the need, as noted above, to reclaim lost gardening and food preservation skills:

There’s a lot of information that actually goes into growing a garden, and this is actually stuff that can start being passed on. I know in our family we haven’t really gardened since grandma and mom, and that was boarding school era. So, it skipped what, two generations? Now, we’re slowing picking it back up again.

One community advisory board member hoped that the project “planted the seed of healthy living” in response to drugs and alcohol, particularly for young people in the WRR. Making an explicit connection to historical trauma, she wondered, “I don’t know how we’re ever going to break that. I know it goes back, way back, generations and generations when it started with the boarding school. And we’re still living that trauma, although we say so much, ‘well what is that?’ We don’t realize that’s still affecting our lives today, and you wonder: how are we ever going to break that?”

### Future

Most participants framed their current gardening experiences with an orientation toward the future, both for themselves and their families in the near and long term. The *future* frame includes themes of *perseverance and expansion; children and grandchildren;* and *visions and hope.*

### Perseverance and expansion

Many participants spoke about continuing to garden for themselves and their families in a practical way in the near term. Even if their garden attempt had presented challenges, most participants demonstrated perseverance and plans to overcome challenges in upcoming seasons. In a narrated video of the extensive grasshopper damage in her garden, one participant stated, “this year is not good, that’s for sure, but all I can do is keep going.” Another participant, whose mother’s death largely prevented her from gardening altogether, described plans for a larger garden and experimenting with different crops for her family in the near future, saying, “I want to try an apple tree. My grandson wants an apple tree, so I’m going to try that and see what happens.”

Participants who enjoyed more successful gardens also spoke of plans for the near future and upcoming gardening season. Numerous participants expressed a desire to expand their gardening skills and knowledge, asking for more information ranging from troubleshooting to food preservation. A couple explained the importance to their family of “learning from your mistakes” in order to plan ahead for different approaches to gardening. In addition to new techniques, many successful gardeners spoke about expanding the size of the garden for their families.

### Children and grandchildren

Much like their focus on family, specifically children and grandchildren, in the present, participants discussed how the garden prepared their children and grandchildren for wellbeing in the future. Many extended their future thinking in terms of ideals for their children and grandchildren throughout their lives and into adulthood with a longer-term future orientation than the immediate plans above, for example:

Showing my kids how to take care of it and letting them grow up to do the same thing.

That way they know when they grow up, this is what you’ve got to do. And, if you want the vegetables, you plant them, watch them, take care of them, feed them.

Learning to eat healthy and the way we were meant to eat, rather than junk food, McDonald’s, Pizza Hut, and all that. We try to eat a lot of vegetables. And it’d be more meaningful for my girls to know how to grow them, so that way they know how to do it when they’re older. Everything I teach them, I want them to hold on to and know when they’re older, when I’m gone.

### Visions and hope

Additionally, participants adopted a broader future orientation focused on visions, hopes, and plans for their gardens, families, and entire people often in direct response to family, food system, and greater societal challenges. One participant discussed her garden in the context of wanting to retire if her adult children would take responsibility for their own children, the grandchildren currently in her care:

These little girls, their mother is always gone. I can’t go anywhere without them, they’ll just, “grandma, where you going?” I try to sneak out the door, and they beat me out the other. If the parents are responsible, I’d like to retire. I’d like to do a big old garden. I want to do flowerbeds.

Another participant explained that his parents had previously farmed grains and, “as we got older, it just kind of went out of style.” His hopes for the future centered primarily on his own children, but also his parents’ wellbeing: “It would be nice to see them pick it back up, because then it would help them emotionally and physically, actually.”

We asked community advisory board members about their visions and hopes for the future both within Growing Resilience and once the project ends. They described more immediate hopes for the board and project itself, but most of them extended their hopes into a broader future for their community and people going forward, much like participants did for their own families:

My hope and vision for this is that there can be better education and awareness getting out there to the younger kids about what processed food is doing to us. I just want that to be a great concern for us as people, for us to be here a long time and to continue our legacy of what we’re supposed to be doing.

### Cross-Generational

Participants explained the gardening experience beyond discrete time-based frames and across more than one generation within their overall stories and even within a single excerpt. For example:

It’s just important for my kids, for my family. Because my dad had diabetes, my grandma and grandpa. I don’t want my kids to have that. I don’t want to have that. I want to actually be able to eat healthy and make sure my family eats healthy.

My daughter and I do the garden together with my grandkids, and I think that’s like what [other participant] was saying: that the most important thing is to pass that on to our families. My grandmother and my great grandmother would also garden in [home state], and my mother had a huge garden.

Participant: “I mean this garden is, for me, it’s to carry on the tradition, especially when my dad’s not here. He’s the one, he’s our leader right now.”

Interviewer: “[Your dad] was saying he remembers his parents and grandparents gardening corn.”

Participant: “Yep, and when we used to live on [street name], we had a garden there, and we’d go along and plant. That was always our family thing. That’s what I want to make for my kids to carry on and know what you got to do. It takes work, but it can be done, and take pride in our land and our seeds and growing here in the nice sunshine.”

### Shared knowledge and memory-making

In addition to describing the gardening experience across multiple generations, participants talked about the transmission of knowledge and the active production of memories across generations and time. One participant explained drawing on memories of her mother to develop present-day gardening skills:

She had a couple gardens when I was younger. She was always planting something, actually. She wouldn’t say, “come here,” but I was just watching her all the time, and I mostly learned from watching. I just remember the things she would do. And then when I would come across these problems, I’d wonder, “what would she think about this or do about this?” And, it really helped a lot trying to get through growing stuff.

Many participants described gardens as explicitly meeting a need for present and future self-reliance, equating those same practices to traditions in the past. One participant thoroughly articulated this concept by connecting his garden to generations past and an unforeseen future:

I’m always thinking about these types of things, because I grew up, my grandma and my grandpa they used to talk a lot about what the old people say. In the future, this is what’s going to happen, foretelling, to prepare yourself. For one, it was preparation as a boy to be a man, this is what a man does, this is how you do it, this is the way that you’re supposed to think about it. And then they also tell you, you’ve got to learn how to do these things, because one day you’re going to need it. You better learn how to eat prairie dog; you better learn how to cook it. One day that might be the only thing you have. Learn now how to eat rock. You better learn how to catch fish and cook it. You better learn how to cut your meat, because one day that’s the only thing you’re going to have to rely on. And one of the things they talked about too, they said one of these days there’s going to be a time when, they talked about a war or some kind of a tragedy, some people think they’re talking about nuclear holocaust. They’re saying you’re going to have to rely on yourself and your skills and your knowledge. And to me, learning how to grow is not that simple as putting the seed in the ground and water it. How much water? How deep should you plant that seed? What kind of dirt?

Finally, participants described their gardens as a means to make memories for themselves and for their families into the future. One participant explained how she wanted to reconnect her cousin with gardening as a way to help her remember her deceased mother who had previously gardened. Many participants hoped to make memories for their grandchildren in the future, including by connecting back to memories of their own grandparents:

I, too, grew up where my grandmother had a garden, and I would be out there working in it, just like my grandkids did too. I really enjoyed that. I mean it’s peaceful. We would both would sit out there, had a bench out there, and they’d come sit out there with me. I look forward to making more memories in my garden.

I had a really good experience this past summer with gardening. And, it really made some good memories for my grandbabies. I think that’s the biggest reason I decided to do gardening…. It really makes me good memories, and I think that’s what I want to leave my grandbabies with is memories, so they can instill that in their kids and carry it on.

## Discussion

Participants consistently contextualized their gardening experience, including health, wellbeing, and food, within family and generational relationships and shared knowledge, practices, and memories. They explained gardening in the present in terms of outcomes for their families, especially children and grandchildren, and how gardens facilitate family teaching and learning, and togetherness. Through making connections to generations in the past, participants recalled parents and grandparents who gardened and aimed to reclaim past knowledge and traditions despite historical trauma. Participants demonstrated a future orientation, explaining immediate plans to persevere and expand for themselves and their families even despite challenges, a longer-term focus on the well-being gardening could provide for their children and grandchildren, and visions of hope for their families and people. Finally, participants connected their gardens across multiple generations, drawing on past, present, and future family relationships at once, including how gardens facilitated shared knowledge and memory-making. Taken together, we suggest that these connections compose what we call *intergenerational resilience.*

**Figure 1.**
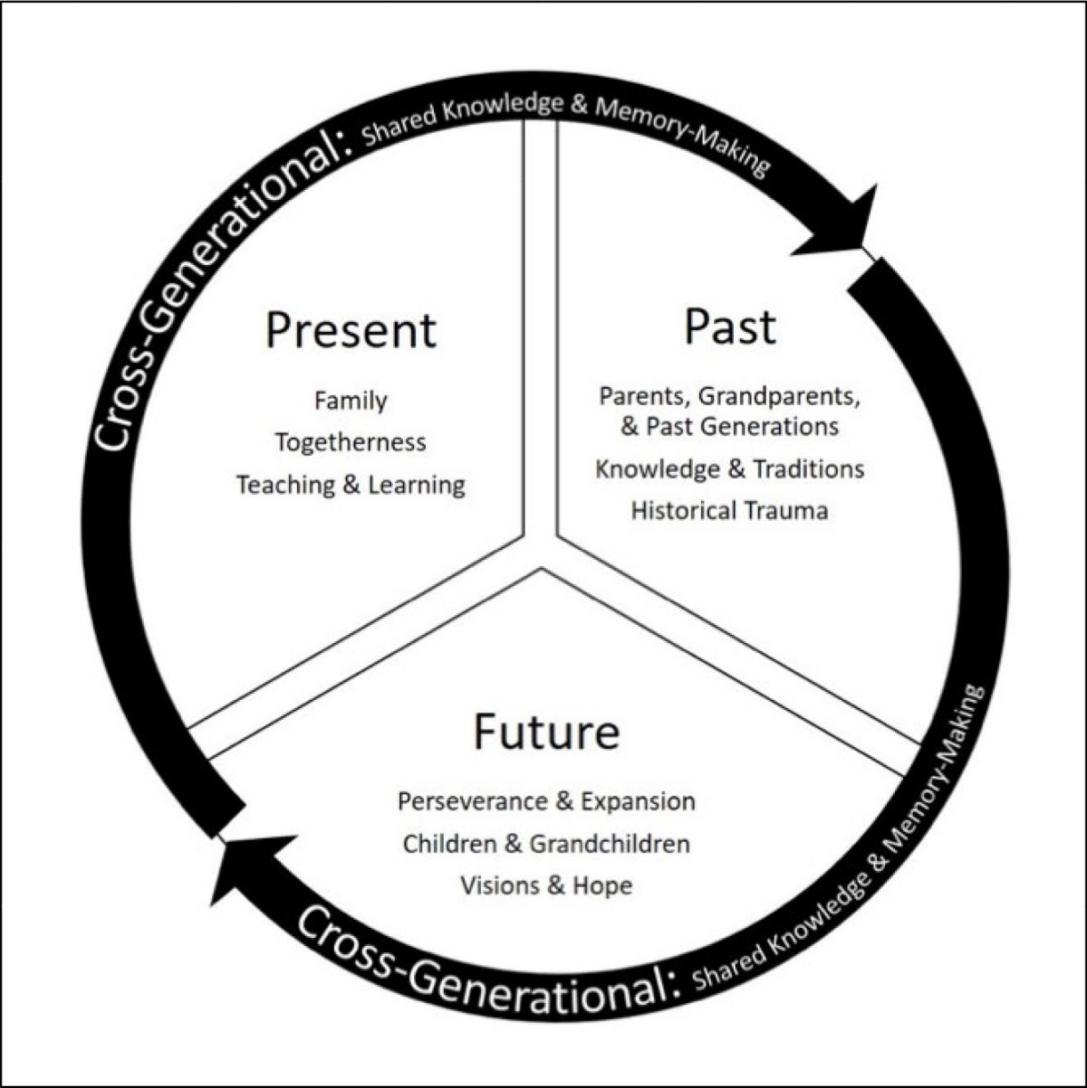
Intergenerational Resilience A cyclical representation of time-based and cross-generational frames, which organize familial and generational themes from participants’ stories.

We present the themes above according to largely chronological concepts of present, past, and future, along with the cross-generational ways participants described their family relationships. Northern Arapaho people who live in the WRR, for example, have broadly adopted these Euro-American concepts of time throughout the process of colonization. Anderson (2011), however, notes that colonization contributed to “dissolving the densely intergenerationally ordered time-space of pre-reservation life” (p. 253). Though our frames follow a Western, linear presentation of time, Figure 1 presents a more culturally appropriate representation of intergenerational resilience, following Arthur and Porter’s (2019) work on re-storying Northern Arapaho food sovereignty with a cyclical paradigm of time.

The saliency with which participants explained their gardening experiences in terms of family in the present—particularly children and grandchildren—was striking. Their focus on teaching and learning and togetherness indicate that they use home gardening not only to produce food and develop practical skills, but also to facilitate important relationships and processes that have little to do with gardening itself. Like the kincentric ecology that undergirds Indigenous food sovereignty, gardens are just one part of an interconnected web of relationships between family members and their environment (Coté, 2016; Salmón, 2000). These findings add a distinctly familial and intergenerational dimension to the characteristics of social networks and knowledge, skills, and learning in community resilience frameworks (Berkes & Ross, 2013).

Participants’ discussions of gaps in past cultural and family gardening knowledge suggest that they understand gardening in Growing Resilience as a resilient response to the colonization and genocide that systematically diminished food sovereignty for people in the WRR (Arthur & Porter, 2019). Furthermore, participants drew on past trauma to explain present barriers to gardening for themselves, their families, and their people, which is consistent with understanding trauma as ongoing structural violence (Kirmayer et al., 2014). Relevant to the connection between resilience and food sovereignty, Walsh-Dilley et al. (2016) remind us that “to build resilience in a particular context, we cannot just look forward but must also look back to understand what social structures and relations of power have created contemporary outcomes” (para 27).

Yet participants readily focused on a resilient reclamation of knowledge and skills from the past, indicative of the process of decolonization and connections to self-determined food practices central to Indigenous food sovereignty (Coté, 2016; Grey & Patel, 2015). One participant’s ability to explicitly relate nourishing plants today to providing a feeling of connection to past generations harkens to another aspect of Indigenous food sovereignty encapsulated in the Cree’s miyupimaatisiium notion of being alive well, which connects to “a rich and complex past” (Adelson, 2000, p. 25), in addition to relationships with the environment. Community resilience frameworks similarly acknowledge that collective memory constructs understandings of the past in a way that can support resilience in the present (Harms, 2012; Vaneeckhaute et al., 2017).

Gardeners expressed the desire to persevere and expand in the near term despite family and environmental challenges, including as a pathway toward wellbeing for children and grandchildren over the longer term. Particularly in Indigenous communities, resilience requires this kind of strength in spite of adversity (Kirmayer et al., 2012). Hopes and visions for a broader future demonstrate that same strength and connect with the foresight and future-orientation key to social-ecological resilience (Westley, Carpenter, Brock, Holling, & Gunderson, 2002). People’s hopes for the future can inform how they direct their present and near-term practices for resilience (Baptista, 2014; Persoon & van Est, 2000).

The themes in the cross-generational frame demonstrate the importance of interconnected relationships supported through a living garden environment across present, past, and future family generations at once. While teaching, learning, and knowledge appear in all frames, the cross-generational frame suggests that the transmission of memories and knowledge is an active process within families. This parallels the O-Pipon-Na-Piwin Cree stories that connect past, present, and a future wherein food “is a source of cultural strength,” which “as *wechihituwin,* represents more than sustenance, it contains stories and memories that can heal the community” (Kamal et al., 2015, p. 570; italics in original). Similarly, we find that gardens provide more than health promotion or reclamation of autonomy over food production (Porter, 2018a; 2018b); gardening can facilitate connections to past, present, and future generations at once. This vibrant approach to generational time is dynamic rather than freezing, erasing, or othering Indigenous people as relics of the past (Fabian, 1983). It draws on relationships across the past and present to inform a more hopeful, relational, and resilient future.

Our findings suggest that gardening facilitates the generational transmission of resilience for Growing Resilience families, which is significant in three main ways. First, intergenerational resilience extends beyond the direct historical trauma response (see Atallah, 2017; Denham, 2008) and applies to home gardening as an Indigenous food sovereignty practice in the WRR. The effects of colonization and genocide are ever-present, including in the food system (Arthur & Porter, 2019; Coté, 2016; Grey & Patel, 2015). Gardens, however, provide space and capacity for families to reinforce their relationships across time in a present context less directly connected with historical trauma (Lester, 2013). Second, we empirically extend the dimensions of intergenerational resilience through the specific ways in which participants draw on relationships and knowledge across the present, past, future, and cross-generationally through gardening to inform resilient practices.

Third, we begin to introduce intergenerational resilience to community resilience frameworks, which has relevance for application in movements for Indigenous food sovereignty. We provide evidence for a dynamic, intergenerational dimension to key community resilience characteristics of people-place relationships; social networks; and knowledge, skills, and learning (Berkes & Ross, 2013) through gardening in the WRR. We conceptualize intergenerational resilience not as a counter-framework to existing community resilience frameworks; rather, it provides a culturally specific dimension of community resilience that is particularly resonant for Indigenous food sovereignty, for which universalized models cannot do justice.

Anthropological concepts of *cultural* resilience are also relevant to understanding the cultural specificity that intergenerational resilience provides. Providing a working definition, Bollig (2014) suggests that cultural resilience is “a set of contextually relative attributes (thoughts, behaviours, knowledges, resources) that intersect across different social networks, scales and institutions within lifetimes, across generations and through historical time” (p. 276). Incorporating generations and historical time pushes community resilience beyond social and ecological networks and processes to a more dynamic, longer-term conceptualization of relationships and culture relevant in Indigenous contexts. As Middleton’s (2010) work with Maidu people demonstrated for political ecology perspectives, social-ecological community resilience approaches can better support Indigenous people by recognizing the centrality of intergenerational trauma—and as we suggest, of intergenerational resilience.

Given the relational kincentric ecologies relevant to other Indigenous food sovereignty efforts (Salmón, 2000), intergenerational resilience could serve as a focal characteristic of social-ecological community resilience approaches in these contexts. In Indigenous food sovereignty efforts, intentionally integrating family into practical strategies of growing, preparing, and sharing food may help people make even more of these practices by generating intergenerational resilience. Sharing family stories of intergenerational relationships, knowledge, memories, and hope may further contribute to resilience development in food sovereignty efforts. In contexts with strong family networks, such as the WRR, CBPR approaches to food sovereignty collaborations with community-based partners should help ensure this approach, drawing the family into sharper analytical focus by helping to shape interventions around the family across generations.

## Conclusion

Families participating in Growing Resilience fostered intergenerational resilience through gardening. We conceptualize this intergenerational resilience not as counter to existing resilience perspectives, but as a culturally specific characteristic or mechanism of community resilience. In this case, the generational transmission of resilience extends beyond the immediate historical trauma response and is particularly applicable to Indigenous food sovereignty. Intergenerational resilience is a strength that Indigenous people and communities may draw and build upon, including in the face of historical trauma.

Community resilience and Indigenous food sovereignty approaches, however, may vary across contexts due to a wide range of Indigenous cultures and also the different effects of colonization and power in unique places (Kamal et al., 2015; Walsh-Dilley et al., 2016). Accordingly, future research should examine if and how intergenerational resilience is relevant in other Indigenous contexts and food sovereignty efforts. Cross-cultural comparisons to non-Indigenous contexts could also provide a better understanding of the role of family across generations in related community resilience and food justice practices. Finally, based on preliminary findings from our data, other social relationships among friends, colleagues, community-based organizations, and broader community structures emerged as important, though they were mentioned less frequently than generational family relationships. Future CBPR in WRR will build on these findings to investigate the importance of family relationships relative to and in concert with other social relationships for health, resilience, and food sovereignty.

In sum, families engaged in the community resilience and food sovereignty practice of home gardening through Growing Resilience fostered and drew strength through intergenerational resilience based not only in relationships and knowledge in the present, but also connections to past and future generations, and even across many generations at once. By focusing on these relationships, gardens and other Indigenous food sovereignty practices may grow resilience more intentionally both for the present and for generations to come.

## Footnotes

1 Prior to foreign intrusion, Eastern Shoshone and Northern Arapaho people were mainly non-agricultural, primarily practicing hunting and gathering, with Northern Arapaho people also originally practicing supplemental crop cultivation (Anderson, 1994; Dorsey & Kroeber, 1997; Loether, 2011). Agricultural practices such as gardening became prominent in WRR-based life due to hunger, the imposition of agricultural materials, and federal promotion of home gardening during World War II. Elders in the WRR specifically recall that gardens provided important subsistence during the boarding school era (Arthur & Porter, 2019). While gardening emerged later and through the colonization process, Eastern Shoshone and Northern Arapaho community members emphasized gardening with Blue Mountain Associates in the Food Dignity project as a way to increase control over their food system and self-determination in keeping with Indigenous food sovereignty.

2 Following best practices suggested by Indigenous thinkers and restorative justice writers to support decolonization and reduced marginalization in writing style, we have opted to present Indigenous words without italicization (see Valandra, n.d.).
